# Supplementary material for: Coated microbubbles swim via shell buckling
Source: Commun Eng. 2023 Sep 7;2:63. doi: 10.1038/s44172-023-00113-z (PMC10955835; doi:10.1038/s44172-023-00113-z)
Supplement: Supplementary file 2 — Supplemental Information (updated title) [file 44172_2023_113_MOESM2_ESM.pdf]

Coated microbubbles swim via shell buckling

Georges Chabouh[1,*], Marcel Mokbel[2], Benjamin van Elburg[3], Michel Versluis[3], Tim Segers[4], Sebastian Aland[2], Catherine Quilliet[1], and Gwennou Coupier[1,*]

[1] CNRS/Université Grenoble-Alpes, LIPhy UMR 5588, Grenoble, F-38401, France.
[2] Technische Universität Bergakademie Freiberg, Akademiestrasse, 609599 Freiberg, Germany
[3] Physics of Fluids Group, Technical Medical (TechMed) Center and MESA+ Institute for Nanotechnology, University of Twente, 7500 AE Enschede, The Netherlands.
[4] BIOS/Lab-on-a-Chip Group, Max Planck Center Twente for Complex Fluid Dynamics, MESA+ Institute for Nanotechnology, University of Twente, Enschede, The Netherlands.
[*] georges.chabouh@sorbonne-universite.fr
[*] gwennou.coupier@univ-grenoble-alpes.fr

Supplementary Note 1

Estimation of the sedimentation velocity

In absence of applied excitation, i.e. in the spherical configuration of the microbubble, we measured the sedimentation direction, which is then set as the new y axis, and velocity. We measured an average velocity of absolute value $v_s = 1.3 \pm 0.1$ μm/s.

The weight of the swimmer can be estimated by balance between drag and buoyancy force. We assume in a first approach that the drag force on the three-body microswimmer is the sum of the drag on each sphere, as demonstrated in[1], therefore its amplitude is equal to $6\pi\eta_f(R_0+r_1+r_2)v_s$, where η_f is the dynamic viscosity of the hosting fluid and the r_i are the radii of the lateral beads.The buoyancy force $\rho_f V_0 g$ is related to the volume of the microswimmer $V_0 = \frac{4}{3}\pi\left(R_0^3 + r_1^3 + r_2^3\right)$ and to the density ρ_f of the fluid. We find a weight $W = 5.74 \pm 0.59$ pN considering the following parameters: $\rho_f = 1000$ kg/m^3, $\eta_f = 10^{-3}$ Pa.s, $R_0 = 4.5$ μm, $r_1 = 2.5$ μm, and $r_2 = 3$ μm.

The buoyancy force in the deflated state must also be estimated. It requires to know the volume of the shell, which cannot be directly measured on the images due to the concave shape of the shell. To this end, we use an estimate of the pressure difference ΔP_{pl} across the shell in the buckled state, which hardly depends on the volume on a wide range[2,3]. A heuristic relationship between this plateau pressure and the mechanical characteristics of the shell, based on numerical simulations, was proposed in[4]:

$$\Delta P_{pl} = \frac{2(1-\nu)\chi}{(1-\nu^2)^{0.75}d}\left(2.34\times 10^{-6} + 0.9(d/R_0)^{2.57}\right), \tag{1}$$

where χ is the in-plane compression modulus, ν the Poisson's ratio and d the thickness of the shell. The modulus χ is usually obtained through various experimental techniques based on acoustic activation of spherical oscillation mode, that takes place at high frequencies (of the order the MHz)[5,6]. Its value depends on the theoretical model used to fit the experimental data ; inconsistencies leading to an apparent dependence of χ on the shell radius were regularly encountered and required the development of deeper models[5,7]. For Sonovue, this modulus is reported to range from 0.024 to 2.61 N/m. A value of 0.5 N/m is usually considered for modeling purposes, value close to that usually encountered for lipidic shells[5,8]. We recently showed that a determination of the elastic modulus based on the observation of the quasi-static compression in the spherical regime yielded values comparable to those determined by acoustic activation[9].

The structure of lipid shells is strongly anisotropic. A proper description of its mechanical properties as a surface can still be reached if one considers instead of the thickness d an effective parameter d_{eff} whose value determines its bending modulus. A previous study on lipidic gel vesicles showed that $\nu = 0.8$ is the value that can best explain the observed shapes[10], while choosing $d_{eff} = 0.1R_0$ (much bigger than the shell thickness) allows to account for the 3-fold bowl shape that is observed on buckled Sonovue[9]. With these parameters, we find $\Delta P_{pl} = 2320$ Pa.

Besides, we assume that the gas obeys a polytropic law, where $P_{int}V^{\kappa}$ is constant, where P_{int} is the inner pressure and V the shell volume. Here κ is the polytropic exponent of the gas. For Sonovue shells that are filled with sulfur hexafluoride, $\kappa \simeq 1.1$[11]. The lower bound for the volume is obtained when the external pressure is maximal, and reads $V_f = V_0\left(\frac{P_{atm}}{P_{max}-\Delta P_{pl}}\right)^{1/\kappa} = 0.86V_0$.

Since the cross section of the buckled shell is essentially a disc along the swimming direction, we assumed that the drag force is unchanged in the buckled state and obtained the corresponding algebraic sedimentation velocity v_b (projection on downward axis $-y$) through

$$v_b = \frac{W - \frac{4\pi}{3}\rho_f g \left(0.86R_0^3 + r_1^3 + r_2^3\right)}{6\pi\eta_f(R_0 + r_1 + r_2)}. \quad (2)$$

We find $v_b = 4.1 \pm 0.2$ μm/s which is ~ 3 times the sedimentation velocity in the spherical state.

Upper and lower bounds for the net displacement in the y direction in one cycle can then be evaluated. This net displacement is equal to the total displacement minus the displacement d_s due to sedimentation, whose time rate differs in the different deformation phases. During the first phase (buckling phase), the shell is going from the initial spherical shape (spherical downward velocity v_s) to a completely buckled shape (buckled downward velocity $v_b > v_s$), the distance d_1 traveled in this phase thus obeys $v_s t_1 < d_1 < v_b t_1$. In the second phase, sedimentation in the buckled shape leads to a displacement $d_2 = v_b t_2$. In the third phase, in a similar way as for the first phase, the displacement is found to obey $v_s t_3 < d_3 < v_b t_3$, while in the spherical configuration, the displacement reads $d_4 = v_s t_4$.

With $t_1 = 0.14$ s, $t_2 = 0.11$ s, $t_3 = 0.14$ s and $t_1 = 0.11$ s, we find that 0.96 μm/cycle $< d_s <$ 1.74 μm/cycle. The total displacement is averaged over 25 cycles and gives: $\sim 0.8 \pm 0.3$ μm/cycle, hence the bounded net displacement of swimming is positive and bounded as: 0.16 μm/cycle $< d_{net} <$ 0.94 μm/cycle.

Supplementary Note 2

Displacement: numerical simulations

Additional data are reported in Fig. 5, where the displacement along time of shells of different stiffnesses χ is plotted for different activating frequencies, with a pressure varying between atmospheric pressure P_{atm} and $P_{atm} + 800$ mbar. Fig. 5d also shows the deformation of the stiffer shell as a function of time for selected frequencies, illustrating the impossibility to buckle for too high frequencies (above 200 kHz).

References

1. Lee, C.-T. & Leith, D. Drag force on agglomerated spheres in creeping flow. *J. aerosol science* **20**, 503–513 (1989).

2. Knoche, S. & Kierfeld, J. Buckling of spherical capsules. *Phys. Rev. E* **84**, 046608 (2011).

3. Quilliet, C. Numerical deflation of beach balls with various poisson's ratios: from sphere to bowl's shape. *Eur. Phys. J. E* **35**, 48 (2012).

4. Coupier, G., Djellouli, A. & Quilliet, C. Let's deflate that beach ball. *The Eur. Phys. J. E* **42**, 1–10 (2019).

5. Chabouh, G., Dollet, B., Quilliet, C. & Coupier, G. Spherical oscillations of encapsulated microbubbles: Effect of shell compressibility and anisotropy. *The J. Acoust. Soc. Am.* **149**, 1240–1257 (2021).

6. Versluis, M., Stride, E., Lajoinie, G., Dollet, B. & Segers, T. Ultrasound contrast agent modeling: a review. *Ultrasound medicine & biology* **46**, 2117–2144 (2020).

7. Dash, N. & Tamadapu, G. Radial dynamics of an encapsulated microbubble with interface energy. *J. Fluid Mech.* **932** (2022).

8. Kawahata, R., Kanagawa, T. & Chabouh, G. Nonlinear ultrasound propagation in liquid containing multiple microbubbles coated by shell incorporating an anisotropy. *Phys. Fluids* (2023).

9. Chabouh, G. *et al.* Buckling of lipidic ultrasound contrast agents under quasi-static load. *Philos. Transactions Royal Soc. A* **381**, 20220025 (2023).

10. Quemeneur, F., Quilliet, C., Faivre, M., Viallat, A. & Pepin-Donat, B. Gel phase vesicles buckle into specific shapes. *Phys. review letters* **108**, 108303 (2012).

11. J. J. Hurly, D. R. D. . M. R. M. Thermodynamic properties of sulfur hexafluoride. *Int. J. Thermophys.* **21**, 739–765 (2000).
